# Socio-demographic characteristics and psychosocial consequences of sickle cell disease: the case of patients in a public hospital in Ghana

**DOI:** 10.1186/s41043-017-0081-5

**Published:** 2017-01-31

**Authors:** Vincent A. Adzika, Franklin N. Glozah, Desmond Ayim-Aboagye, Collins S. K. Ahorlu

**Affiliations:** 1 Department of Psychology, Regent University College, Accra, Ghana; 2 Department of Epidemiology, Noguchi Memorial Institute for Medical Research, University of Ghana, Accra, Ghana

**Keywords:** Sickle cell disease, Quality of life, Anxiety, Depression, Socio-demographic characteristics, Ghana

## Abstract

**Background:**

Sickle cell disease (SCD) is of major public health concern globally, with majority of patients living in Africa. Despite its relevance, there is a dearth of research to determine the socio-demographic distribution and psychosocial impact of SCD in Ghana. The objective of this study was to examine the socio-demographic distribution and psychosocial consequences of SCD among patients in Ghana and to assess their quality of life and coping mechanisms.

**Methods:**

A cross-sectional research design was used that involved the completion of questionnaires on socio-demographic characteristics, quality of life, coping mechanisms, anxiety and depression. Participants were 387 male and female patients attending a sickle cell clinic in a public hospital.

**Results:**

Results showed that majority of the patients were single, female, less than 39 years old and had attained secondary school level of education or less. Also, patients were more satisfied by the presence of love, friends and relatives as well as home, community and neighbourhood environment. While pains of varied nature and severity were the major reasons for attending hospital in SCD condition, going to the hospital as well as having faith in God was the most frequently reported mechanisms for coping with an unbearable SCD attacks. Results of multiple regression analysis showed that some socio-demographic and quality of life indicators had strong associations with anxiety and/or depression.

**Conclusions:**

It is recommended that a holistic intervention strategy incorporating psychosocial dimensions should be considered in the treatment and management of SCD.

## Background

Sickle cell disease (SCD) is a genetic disorder found in individuals who have inherited an abnormal gene in the haemoglobin from either parent [1]. The disorder follows a more severe clinical course among patients in Africa than in those outside Africa [2]. In sub-Saharan Africa, about 300,000 infants are born with major haemoglobin disorders and about 2% of all children have SCD [3]. The frequency of the trait is between 15 and 30% in West Africa and 2% in Ghana [4]. It has been reported that the Komfo Anokye Teaching Hospital in Kumasi, Ghana, currently manages 6000 newborn babies diagnosed with SCD and this represents the largest number of newborn babies with the blood disorder being taken care of under one facility in the world [5]. Managing SCD among this large number of individuals presents a huge burden on the financial resources of Ghana, implying that SCD is a significant public health concern in Ghana. Extensive SCD research has been conducted in Ghana spanning several decades [6–8], but more research is needed to explore the socio-demographic distribution and psychosocial consequences of SCD on patients. The socio-demographic distribution of patients and psychosocial consequences of SCD is however not well documented. Therefore, it is imperative to systematically document and ascertain the present socio-demographic distribution and the psychosocial consequences of SCD on patients.

There is a pattern in the socio-demographic distribution of SCD globally. For instance, a previous study found that majority of their sample were females, had high school education, were between 25 and 34 years old and were single [9]. Levenson et al. [10] found that most of their samples were females, had high school education, were from 25 to 34 and were single. Amaral et al. [11] also found that among their sample, the highest frequency was recorded for patients who were married, were females, were between 30 and 39 years old and had attained high school education. Finally, Asnani et al. [12] reported that the mean age of the participants in their study was 30 years old, most of whom were females and most had attained secondary/high school level of education.

In terms of the psychosocial consequences of SCD, several studies have found that SCD, which is a life-threatening disease, has severe psychosocial consequences on individuals [13–15]. Accordingly, individuals who have chronic illnesses such as SCD are three to four times more likely to develop various mental health disorders [16]. SCD patients are also at risk of maladjustment to life in several functional areas including emotional and behavioural problems, poor self-concept and interpersonal functioning and limited athletic abilities [17, 18]. Laurence et al. [19] found that among African-American adults, the odds of having significant depressive symptoms were much higher for those with severe forms of clinical SCD. Also, the prevalence of depression has been found to be 21.6% in SCD patients and 9.4% in controls in a Jamaica sickle cell cohort study [12]. African-American adults with SCD experience higher levels of depression (26%) or depressive symptoms (32%) compared to 9.5% in the rest of the population [20]. Furthermore, it has been found that SCD patients with severe and extremely severe anxiety report significantly higher proportion of vaso-occlusive crisis [21]. Thus, screening for depression is important to ensure that the diagnosis and treatment of depression is properly coordinated and identified [20].

Health-related quality of life (QOL) refers to the effects an illness has on the subjective well-being of patients [18]. This includes children with SCD who also have lower daily functioning capacity and physical limitations as a result of SCD [22]. Measuring QOL has therefore become increasingly important in evaluating interventions, assessing prognostic factors, comparing therapies and allocating resources [23]. Dampier et al. [24] found that there is a substantial impairment of health-related QOL in adults with SCD. The pain associated with the SCD crises and frequent hospitalizations has a significant impact on the QOL of patients [25]. Thus, pain has been reported to be the predominant reason why SCD patients visit the hospital [26].

Individuals with SCD use various mechanisms to cope with the disease, which largely includes pain management. Coping mechanism involves constantly changing cognitive and behavioural efforts to manage internal or external demands on individuals’ personal resources [27]. Attending a hospital or clinic and taking medication are known to be some of the means of coping with SCD crisis [26]. In addition, religious beliefs mitigate the uncomfortable feelings that are associated with SCD. For example, through religious beliefs, individuals are able to cope with personal difficulties and stress by seeking strength from God or a supernatural being through prayer and meditation [28]. This also involves associating with other believers in fellowship that may lead to renewed and deeper faith in God or a supernatural being [29, 30].

In spite of these, not much is known about the present socio-demographic distribution and psychosocial consequences of SCD in Africa and Ghana in particular. Much is known about the psychosocial response of other diseases such as diabetes than for SCD in Ghana. Furthermore, although treatment advances greatly improve the quality of life as well as the lifespan of patients, exploring the present treatment and quality of life outcomes would help in the management of the patient. The purpose of this study was to examine the present socio-demographic distribution and psychosocial consequences of SCD and to incorporate these into the management of SCD in Ghana.

## Methods

### Population and sample

A cross-sectional survey design was used in the study. The population consisted of SCD patients at a sickle cell clinic in a major hospital in Accra. About 24,010 patients attend this hospital in a year, 100 to 120 patients (both old and new) attending daily. A simple random sampling technique was used to select patients, from a list, who attended the hospital within the 2-month period of data collection. Those willing and able to participate in the study and signed an informed consent from were recruited for the study. In all, 387 patients participated, comprising of 180 males and 204 females with a mean age of 24.34 (SD = 6.59) years.

### Measures

Four different assessment instruments were used in this study. These are the Beck Depression Inventory, the Beck Anxiety Inventory, the quality of life index, and the structured clinical interview. These measures were appropriate for SCD patients in Ghana. Although these measures were developed outside Ghana, cross-cultural studies have found that the Beck Depression Inventory (BDI), for example, is satisfactory for measuring cross-cultural depression [31]. A cross-cultural study, that included Ghana, found that there were no significant differences in BDI scores among countries [32]. Regardless of this, in order to ascertain whether these measures assess what they purport to assess in the present sample, we conducted guided focus group discussions with eight participants in a group in a total of five groups. We found that participants’ views, knowledge and understanding of depression, anxiety and quality of life did not differ much from the items on the tools. In respect of this, the tools were used in their original form without modifications.

#### Depression

The BD) [33] was used to assess depression. This is a 21-item multiple-choice self-report inventory, widely used in screening for both the presence and severity of depression. The BDI total score is the sum of the ratings for the 21 symptoms. Each symptom is rated on a 4-point scale ranging from 0 to 3 with total scores ranging from 0 to 63. Higher scores indicate more severe depression. On scale, 0 to 9 represents minimal depression; 10 to 16 indicates mild depression; 17 to 29, moderate depression; and 30 to 63, severe form of depression. If a patient scored very high on the BDI (especially on item number 9 regarding suicidal thoughts), we drew the attention of their personal doctor in the hospital for further action. Among adult SCD patients, Gallagher et al. [34] found a Cronbach’s alpha of 0.91 for the BDI. In the present study, a Cronbach’s alpha of 0.86 was found for the BDI.

#### Anxiety

The Beck Anxiety Inventory (BAI) [35] is a 21-question multiple-choice self-report inventory that is used for measuring the severity of anxiety. The questions in this instrument are about common symptoms of anxiety the individual has had during the past 4 weeks. These symptoms include numbness tingling, sweating not as a result of hot weather or exercise and fear of pending danger. The BAI total score is the sum of the ratings for the 21 symptoms. Each symptom is rated on a 4-point scale, 0–7 represents minimal anxiety; 8–15, mild anxiety; 16–25, moderate anxiety; and 26–63, severe anxiety. The BAI is psychometrically sound with Cronbach’s alpha ranging from 0.92 to 0.94 [35]. In the present sample, the Cronbach’s alpha was 0.74.

#### Quality of life

The QOL index is a measure of an individual’s quality of life through self-report of the importance they attach to each of 16 life domains on a 3-point rating scale as well as their current satisfaction with each domain on a 6-point rating scale [36]. The importance scores are multiplied by the satisfaction scores for each domain. These scores are then added to determine the overall state of quality of life for the individual. This measure is quick to obtain and to compare with normed values from the community from which changes in individuals in the course of therapy can also be determined and tracked. Higher scores indicate higher quality of life [37]. Cronbach’s alpha for the present sample was 0.88.

#### Clinical interview

The structured clinical interview (SCI) is an instrument that helps a clinician to formulate questions to fit the patients understanding, to ask additional questions that clarify ambiguity, to challenge inconsistencies in responses provided and to determine the seriousness of symptoms presented. It is subjective and limited to diagnostic evaluations, research and the training of health professionals. The SCI used in the present study sought information about major complaint, presenting problems, psychosocial history, individual medical history, family medical history, previous treatment and current medical treatment. It was also designed to elucidate the coping strategies of the patient.

### Statistical analysis

Prior to sampling participants, G*power [38] was used to estimate the required sample size given alpha of 0.05, 95% power and effect size of 0.06. The resulting sample size was 336 participants. The Statistical Package for the Social Sciences (SPSS version 18) was used to conduct the statistical analyses. Descriptive statistics were used to estimate the frequency of the major psychosocial variables in the study and also to examine the socio-demographic distributions of these variables. One-way analysis of variance was used to examine group differences in the socio-demographic variables on anxiety and depression. Post hoc analyses with Bonferroni test for significance was used when there were significant main effects in the independent variables. Multiple regression analyses were conducted with the socio-demographic characteristics and quality of life indicators as independent variables and anxiety and depression as dependent variables, respectively. Statistical estimates were assessed at the 0.05 level of significance.

## Results

### Socio-demographic characteristics of the sample

Table 1 shows the socio-demographic characteristics of the sample. There were more females than males. Majority of the participants were between the ages of 20 and 29 years, and most of them were single. Close to a third of the participants had a secondary or vocational school level of education.

**Table 1:** Socio-demographic characteristics of respondents

Characteristics	Number	Percent
Age group (*n = 385*)		
<20	85	21.30
20–29	142	36.88
30–39	95	24.68
40–49	29	7.53
50–59	20	5.19
60+	14	3.64
Gender (*n = 384*)		
Male	180	46.88
Female	204	53.12
Educational level (*n = 382*)		
No education/primary	28	7.33
Junior high/form 4	96	25.13
Secondary/vocational	166	43.46
Tertiary	92	24.08
Marital status (*n = 372*)		
Single	312	83.87
Married	34	9.14
Divorced	26	9.68

### Socio-demographic characteristics and anxiety and depression

Table 2 shows the results of the one-way ANOVA conducted separately for anxiety and depression by age group, level of education and marital status. Results indicate that there were significant age group differences in depression scores but not in anxiety scores. Post hoc analyses using Bonferroni test for significance in depression scores indicated that mean depression scores was significantly higher in the 40–49 years age group compared to the other age groups. Also, there was a significant relationship between depression scores and education, but not with anxiety scores. Post hoc analysis using least significant differences showed that mean depression scores were significantly higher for those with primary level of education than for the other levels of education. Finally, one-way ANOVA results showed that there were no relationships between either anxiety and depression and marital status (Table 2).

**Table 2:** One-way ANOVA showing socio-demographic characteristics differences in anxiety and depression

Characteristics	Anxiety				Depression			
	*M*	SD	*df*	*F*	*M*	SD	*df*	*F*
Age group								
<20	24.47	10.00	(5, 386)	1.65	15.37	8.67	(5, 386)	4.86**
20–29	26.55	10.54			15.86	8.15		
30–39	19.33	10.75			15.19	7.49		
40–49	37.75	2.23			24.50	4.36		
50–59	17.00	6.40			10.43	5.62		
60+	11.00	0.00			0.00	0.00		
Educational level								
No education/primary	29.56	10.26	(3, 386)	1.69	21.67	8.77	(3, 386)	5.24**
Junior high/form 4	27.00	11.55			13.00	9.24		
Secondary/vocational	25.88	10.10			15.70	7.65		
Tertiary	23.13	11.46			13.84	8.25		
Marital status								
Single	25.26	10.64	(2, 386)	1.06	15.49	8.11	(2, 386)	1.89
Married	26.92	11.41			17.75	9.39		
Divorced	36.00	0.00			26.00	0.00		

**p < 0.01

### Indicators of quality of life in patients

**Fig. 1:**
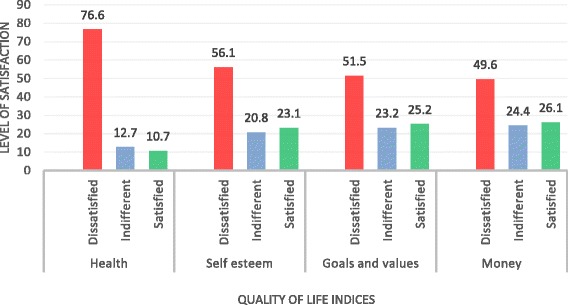
Quality of life indicators: health, self-esteem, goal and values and money. Four qualities of life indicators: health, self-esteem, goal and values and money. The *x*-axis represents quality of life indices, and the *y*-axis represents the level of satisfaction with each quality of life index. *Red*, *blue* and *green bars* represent dissatisfaction, indifference and satisfaction with quality of life, respectively

**Fig. 2:**
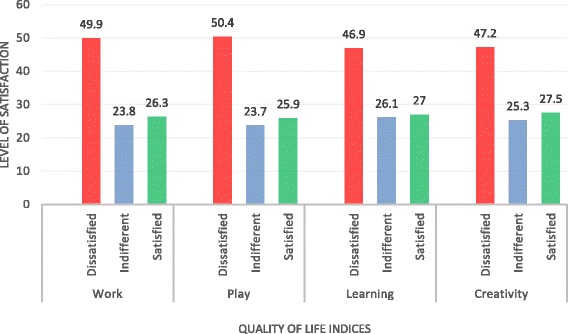
Quality of life indicators: work, play, learning and creativity. Four qualities of life indicators: work, play, learning and creativity. The *x*-axis represents quality of life indices, and the *y*-axis represents the level of satisfaction with each. *Red*, *blue* and *green bars* represent dissatisfaction, indifference and satisfaction with quality of life, respectively

The patients were asked to indicate their level of satisfaction or dissatisfaction in 16 different dimensions of quality of life. The patients were generally dissatisfied with their quality of life, and this was highest for health, self-esteem, goals and values as well as money (Fig. 1). This is even more compelling given that the proportion of indifference in satisfaction have similar levels. The levels of dissatisfaction with quality of life and/or satisfaction were similar across the four quality of life dimensions—work, play, learning and creativity (Fig. 2). The level of indifference was also similar across these four dimensions of quality of life.

**Fig. 3:**
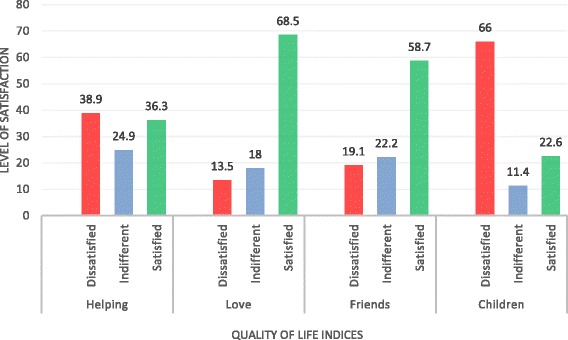
Quality of life indicators: helping, love, friends and children. Four qualities of life indicators: helping, love, friends and children. The *x*-axis represents quality of life indices, and the *y*-axis represents the level of satisfaction with each. *Red*, *blue* and *green bars* represent dissatisfaction, indifference, and satisfaction with quality of life, respectively

**Fig. 4:**
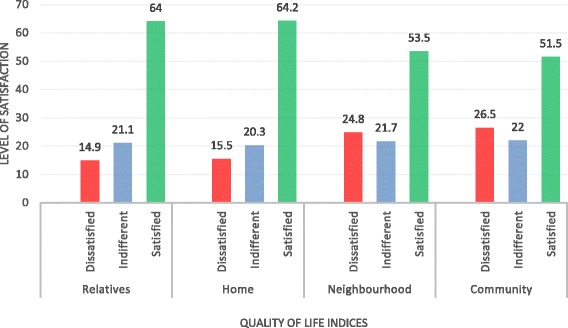
Quality of life indicators: relatives, home, neighbourhood and community. Four quality of life indicators: relatives, home, neighbourhood and community. The *x*-axis represents quality of life indices, and the *y*-axis represents the level of satisfaction with each. *Red*, *blue* and *green bars* represent dissatisfaction, indifference and satisfaction with quality of life, respectively

The patients reported they were more satisfied than dissatisfied with the love received and with friends. They were however more dissatisfied with their children and helping relationships (Fig. 3). Also, the patients reported that they were more satisfied with their relatives, home, neighbourhood and community, indicative of their sense of belonging to the family or community (Fig. 4). Results of these 16 indicators of quality of life suggest that the patients were generally dissatisfied with their quality of life with respect to the first eight domains (Figs. 1 and 2) and more satisfied with the last eight (Figs. 3 and 4).

### Clinical interview indicators

Table 3 shows the reasons why patients visited the hospital. The highest reported symptoms resulting in hospitalisation were severe bodily pains and malaria, and the least were alleged typhoid fever and rheumatism.

**Table 3:** Major complaints/reasons for visiting the hospital

Complaints	Number	Percent
Bodily pains/back pains	159	45.6
Malaria	57	16.3
Check up	42	12.0
Others (e.g. wound, mouth sore, cough, swollen feet)	23	6.6
Fever/vomiting	22	6.3
Dizziness	13	3.7
Abdominal pains/menstrual pains	8	2.3
Weakness	8	2.3
Headache	6	1.7
Crisis	5	1.4
Rheumatism	3	0.9
Typhoid fever	3	0.9

Seeking help at the hospital was the main coping mechanism whenever patients had a crisis (Table 4). However, many participants relied on their personal touch with God as well as keeping to medication regime.

**Table 4:** Coping mechanisms used to manage crisis among respondents

Coping mechanism	Number	Percent
Going to hospital/clinic	224	64.9
Take my medication, drugs, etc.	155	44.9
Believe (have faith) in God	114	33.0
Avoid people/withdraw from social activities/stop work	11	3.2
Try to avoid it/take my mind off	11	3.2
Others, e.g. psyche myself, encourage myself	11	3.2
Bath and sleep/relax	8	2.3
Call on friends/relatives	6	1.7
Take water/fruits	5	1.4
Go to church	4	1.2

### Socio-demographic characteristics, quality of life and anxiety

Multiple regression analysis was used to test if the socio-demographic characteristics and quality of life indicators significantly predicted patients’ anxiety. The results indicated that the predictors explained 36% of the variance. Level of education, quality health, number of children and a conducive neighbourhood significantly predicted anxiety (Table 5). This indicates that higher levels of education, satisfaction with health, availability of children and quality of neighbourhood mitigate anxiety levels.

**Table 5:** Multiple regression analysis showing socio-demographic characteristics and quality of life indicators on anxiety

	*B*	SE *B*	*β*	*F*	*R* ^2^
(Constant)	24.41	2.77		8.04**	0.36
Sex	1.11	0.77	0.05		
Age	−.08	0.07	−0.06		
Education	−1.56	0.48	−0.15**		
Marital status	0.94	1.63	0.03		
Health	−0.84	0.22	−0.26**		
Self-esteem	−0.36	0.34	−0.12		
Goals and values	0.21	0.40	0.07		
Money	−0.26	0.40	−0.08		
Work	−0.09	0.49	−0.03		
Play	−0.63	0.46	−1.20		
Learning	−0.85	0.51	−0.27		
Creativity	0.68	0.50	0.21		
Helping	0.17	0.19	0.06		
Love	0.05	0.26	0.02		
Friends	0.06	0.25	0.02		
Children	0.68	0.21	0.21**		
Relatives	0.45	0.35	0.17		
Home	0.26	0.37	0.10		
Neighbourhood	−0.78	0.39	−0.29*		
Community	0.18	0.37	0.06		

*p < 0.05; **p < 0.01

### Socio-demographic characteristics, quality of life and depression

Results of multiple regression analysis showed that the predictors explained 21% of the variance. Age, health, money, learning and creativity significantly predicted depression (Table 6). This indicates that getting older, satisfaction with health and ability to learn are negatively associated with depression. Also, an individual’s satisfaction with their creativity and money was positively associated with depression.

**Table 6:** Multiple regression analysis showing socio-demographic characteristics and quality of life indicators on depression

	*B*	SE *B*	*β*	*F*	*R* ^2^
(Constant)	38.25	4.07		4.72**	0.21
Sex	0.93	1.13	0.04		
Age	−0.26	0.10	−0.15**		
Education	−0.83	0.70	−0.06		
Marital Status	3.07	2.40	0.07		
Health	−0.84	0.32	−0.19**		
Self-esteem	−0.43	0.51	−0.11		
Goals & Values	−0.55	1.58	−0.13		
Money	1.23	1.58	0.28*		
Work	−0.06	0.71	−0.02		
Play	−0.22	0.68	−0.05		
Learning	−2.84	0.75	−0.65***		
Creativity	1.78	0.74	0.41*		
Helping	0.29	0.27	0.08		
Love	−0.41	0.39	−0.11		
Friends	−0.21	0.37	−0.06		
Children	0.40	0.31	0.09		
Relatives	0.75	0.51	0.20		
Home	0.18	0.54	0.05		
Neighbourhood	−0.06	0.58	−0.02		
Community	−0.28	0.55	−0.07		

*p < 0.05; **p < 0.01; ***p < 0.001

## Discussion

The aim of this study was to examine the socio-demographic distribution and psychosocial consequences of SCD among patients in Ghana. The results showed that majority of the participants were females, between 20 and 39 years old, had secondary/vocational school level of education and were single. These findings are generally consistent with findings of previous studies that have examined the socio-demographic characteristics of SCD patients in other parts of the world [9–12]. Life expectancy of people with SCD is reduced considerably, especially among children and older people. For example, the incidence of infarctive cerebrovascular accident is lowest in patients between 20 and 29 years of age and higher in children and older patients [39]. This indicates that SCD-related mortality is likely to be higher among children and older people and less in those from 20 to 29, which partly explains why majority of SCD patients were between 20 and 39 years old.

The results also showed that most of the participants were females. Gender role socialisation and expectations may have accounted for more females reporting SCD and its associated complications. In Ghanaian culture, males are expected to show bravery, strength and endurance during times of crisis and are expected to be the bread winners, to be leaders in their family and to endure pain [40]. This is corroborated by the fact that females generally report or complain more than males when they are suffering from different types of illnesses [41].

Majority of the participants in this study had attained secondary/vocational school education or less, consistent with findings of previous studies [10, 12]. The reason why lower levels of education are associated with higher numbers of SCD may be because education provides the knowledge and information needed in the management of, the treatment for and effectively coping with SCD. It has been asserted that many children with SCD are underachievers in school [42], which may influence school absenteeism—a significant problem for adolescents with SCD [43]. By deduction, underachievement and absenteeism may result in school dropout which impacts the socio-economic circumstances of individuals which in turn has a negative effect on the ability to cope with SCD. Neighbourhood socio-economic distress has been found to predict quality of life, and living in a distressed neighbourhood predicts diminished health-related quality of life in SCD patients [18]. This indicates that educated individuals are more likely to be better informed about SCD and its management and outcomes. Thus, this may explain why majority of SCD patients are likely to have attained lower levels of education.

In relation to the findings on marital status, majority of the participants reported that they were single and this is compatible with previous findings [10]. It is plausible to posit that patients who are married are more likely to perceive or receive support from their spouse with regard to management of and treatment options for SCD. Furthermore, single patients may be reluctant to get married because of fear of giving birth to children with the sickle cell trait or thought that they will be a burden on their prospective spouse. It has been reported that more than 60% of the participants in a sickle cell study were likely to cancel at-risk marriages [44]. Therefore, more single individuals compared to their married counterparts are likely to report or be patients of SCD.

In relation to quality of life, the results of the present study showed that patients were more satisfied with their ability to work, learn, play, be creative and be happy with friends, which is consistent with findings of previous studies. For example, Dampier et al. [24] found that there is substantial impairment of health-related quality of life in adults with SCD who are in severe pain, hospitalised and are receiving blood transfusions. Moreover, the factors that threaten the survival of SCD patients have a negative impact on the quality of life of patients [25]. Also, it has been found that SCD patients have lower daily functioning abilities and general physical limitations [22]. This suggests that the constitution of the indicators of quality of life is universal, and therefore, it is not surprising that what Ghanaian patients construe as quality of life is similar to that of patients in other countries or cultures.

Our findings are similar to those in previous studies that also found that the main reason why SCD patients visit the hospital was because of pain. Bloom [26], for example, found that abdominal and muscular pains were the main complaints presented by patients reporting at the hospital. This is not surprising because pain has been described as the clinical hallmark of SCD with painful vaso-occlusive episodes being common, debilitating and a medical emergency [45]. It is thus expected that SCD patients would visit the hospital or clinic for treatment of this pain. As expected, patients in the present study visited the hospital or clinic and resorted to taking their medication during SCD crisis. In addition, patients went to church to seek help from a supernatural being during SCD crisis—a significant number depended on their personal faith in God to cope with an unbearable SCD condition. It has been asserted that religious beliefs and going to a place of worship have a positive impact in coping and also seen as a hopeful approach when individuals have difficulties with their health [29], similar to the effects and role of praying [28]. In Africa and in Ghana, for that matter, a large proportion of the people are religious so they rely on their beliefs as well as prayers in times of difficulty, including SCD crisis, indicating that religious beliefs could potentially serve as an effective coping mechanism by SCD patients.

With regard to the association between quality of life and anxiety and depression, good health and ability to learn had negative associations with depression. Creativity and money on the other hand were positively associated with depression. It is known that depression has a positive association with functional impairment and a negative association with quality of life [46, 47]. Low levels of family income have also been found to have a negative association with depressive symptomatology [48]. Furthermore, patients with low family income are more likely to be depressed than those endowed financially [49, 50]. As previously stated, higher levels of education (income) would mitigate the deleterious effects of the psychosocial consequences of SCD—anxiety and depression. Patients with higher education or income are, thus, in a position to have information on the best means to manage SCD and to afford various treatment options.

This study has some limitations that is worth mentioning in order to serve as a guide for the design and conduct of future studies. First, given the relatively large number of patients who visit the hospital annually, selecting a much larger sample would have increased the generalisability of the findings of the study. Nonetheless, the sample size used for this study was enough with respect to statistical requirements. Second, the study would have benefitted from a more objective assessment of quality of life to corroborate the subjective assessment of satisfaction or dissatisfaction. Also, self-report measures, as was used in the study, may cause systematic measurement errors (methods variance), and responses given by participants may not have been accurate. Finally, not including a control group as a basis to compare differences and relationships among the study variables limits the ability to generalise the findings therefore interpretation of the results should be done with due cognisance to this fact.

## Conclusions

This preliminary study reveals that socio-demographic characteristics and quality of life play a major role in SCD and these have serious psychosocial consequences, especially anxiety and depression, on SCD patients. These factors—age, levels of education (income), gender and social relations (marital status)—should be considered seriously in the management of SCD to facilitate healing and restoration of quality of life. More of such studies are needed in Ghana to gain a better insight into the impact of the psychosocial life of SCD patients and their families.

## Abbreviations

BAI: Beck Anxiety Inventory
BDI: Beck Depression Inventory
QOL: Quality of life
SCD: Sickle cell disease
WHO: World Health Organisation
